# Age-group determination of living individuals using first molar images based on artificial intelligence

**DOI:** 10.1038/s41598-020-80182-8

**Published:** 2021-01-13

**Authors:** Seunghyeon Kim, Yeon-Hee Lee, Yung-Kyun Noh, Frank C. Park, Q.-Schick Auh

**Affiliations:** 1 Robotics Laboratory, Department of Mechanical and Aerospace Engineering, Seoul National University, Seoul, Korea; 2 Department of Orofacial Pain and Oral Medicine, Kyung Hee University Dental Hospital, #26 Kyunghee-daero, Dongdaemun-gu, Seoul, 02447 Korea; 3 Department of Computer Science, Hanyang University, Seoul, Korea

**Keywords:** Anatomy, Biomarkers, Medical research, Mathematics and computing

## Abstract

Dental age estimation of living individuals is difficult and challenging, and there is no consensus method in adults with permanent dentition. Thus, we aimed to provide an accurate and robust artificial intelligence (AI)-based diagnostic system for age-group estimation by incorporating a convolutional neural network (CNN) using dental X-ray image patches of the first molars extracted via panoramic radiography. The data set consisted of four first molar images from the right and left sides of the maxilla and mandible of each of 1586 individuals across all age groups, which were extracted from their panoramic radiographs. The accuracy of the tooth-wise estimation was 89.05 to 90.27%. Performance accuracy was evaluated mainly using a majority voting system and area under curve (AUC) scores. The AUC scores ranged from 0.94 to 0.98 for all age groups, which indicates outstanding capacity. The learned features of CNNs were visualized as a heatmap, and revealed that CNNs focus on differentiated anatomical parameters, including tooth pulp, alveolar bone level, or interdental space, depending on the age and location of the tooth. With this, we provided a deeper understanding of the most informative regions distinguished by age groups. The prediction accuracy and heat map analyses support that this AI-based age-group determination model is plausible and useful.

## Introduction

Accurate age-group estimation of an individual is extremely important in forensic dentistry and for various medico-legal purposes. Age-group estimation is the process of determining a person’s age group based on biometric features^1^. In particular, as immigration and the number of refugees around the world increase, the demand for rapid age estimation is growing^2^. In addition, age group classification can help in making robust, but rapid judgments, and has many applications in fields such as homeland security, passport services, statistical analysis of group-wise age distributions, and forensic science. As an active research area, machine-based age group estimation algorithms as well as human perception-based methods for age estimation have been reported in the literature^3,4^. Despite significant advances and ample work in related research areas, a definitive and effective method for age-group determination for all ages has not yet been achieved.

Teeth are considered a reliable biological marker of aging as they are highly durable, resistant to putrefaction, fire, and chemicals. Compared with an assessment of the ossification stage of the medial clavicular epiphysis in bone development, dental development, and eruption provides a reliable indication of chronological age^5^. Each state of dental mineralization is hardly affected by environmental or hormonal variations. Commonly utilized dental methods in children and adolescents include the analysis of dental development, tooth eruption, the mineralization of the tooth crowns and roots^6,7^, and open apices of the root^8^. For adults, other methods using the tooth-coronal index^9^, the level of the alveolar bone^10–12^, and the pulp/tooth area ratio^13,14^ have been reported; however, the accuracy is lower than that for children and adolescents. With increasing age, alveolar bone levels and pulp to tooth ratios tend to decrease, but the error range using direct measurements of the first molar is 8.84 years^15,16^. Dental age estimation displays unique challenges owing to complex variations in the shapes and sizes of teeth, both within and across people. In addition, previous methods had a major drawback of focusing only on partial features of teeth with large error ranges in age estimation.

Considering the high potential for errors and bias associated with conventional age estimation methods, we hypothesized that the elimination of subjective aspects and automatic performance of age-group estimation would result in improved performance. Throughout the last decade, continuous efforts to improve the accuracy of AI-based estimations, one of which is the incorporation of deep learning algorithms, have been made. Convolutional neural networks (CNNs) have been reliably used for various problems in the dental field, using dental X-ray images^17–19^. The main difference between CNNs and conventional individual feature-based methods is that CNNs perform end-to-end learning and extract a set of relevant features directly from raw data, without human intervention. Although many methods have been derived and modified based on the Demirjian method, which was first described in 1973^20^, it allows for subjective judgment by observers, and its wide error range is still an issue. As human-engineered procedures are not required with CNNs, an AI system significantly reduces the workload of human interpreters or observers in dental age prediction^21^. In addition because CNNs autonomously learn a holistic feature set from data, they exhibit robust performance with a large amount of data.

This study was designed to propose a novel deep learning system that provides a fully automated model for age-group determination using panoramic radiographs of the first molar. To the best of our knowledge, this is the first study of dental age-group estimation based on CNNs that directly uses images of first molars. Using CNN allows us to grasp the entire radiographic feature of panoramic radiographs, not only the partial radiographic characteristics of the first molar. As AI-based approaches can process large amounts of data, it is expected that our method will be useful for verifying age groups in mass disasters and refugee issues, and various anthropological and legal issues. Furthermore, our method may reduce the shortcomings of previous methods by minimizing errors caused by subjective experiences and increasing diagnostic accuracy and speed. Through a heat map using a gradient-weighted class activation mapping (Grad-CAM) algorithm, we provide visualizations of the information and recognized tooth and alveolar bone regions in the X-ray image patches extracted from panoramic radiographs used by the CNNs for the determination of age groups.

## Materials and methods

### Study population

**Figure 1:**
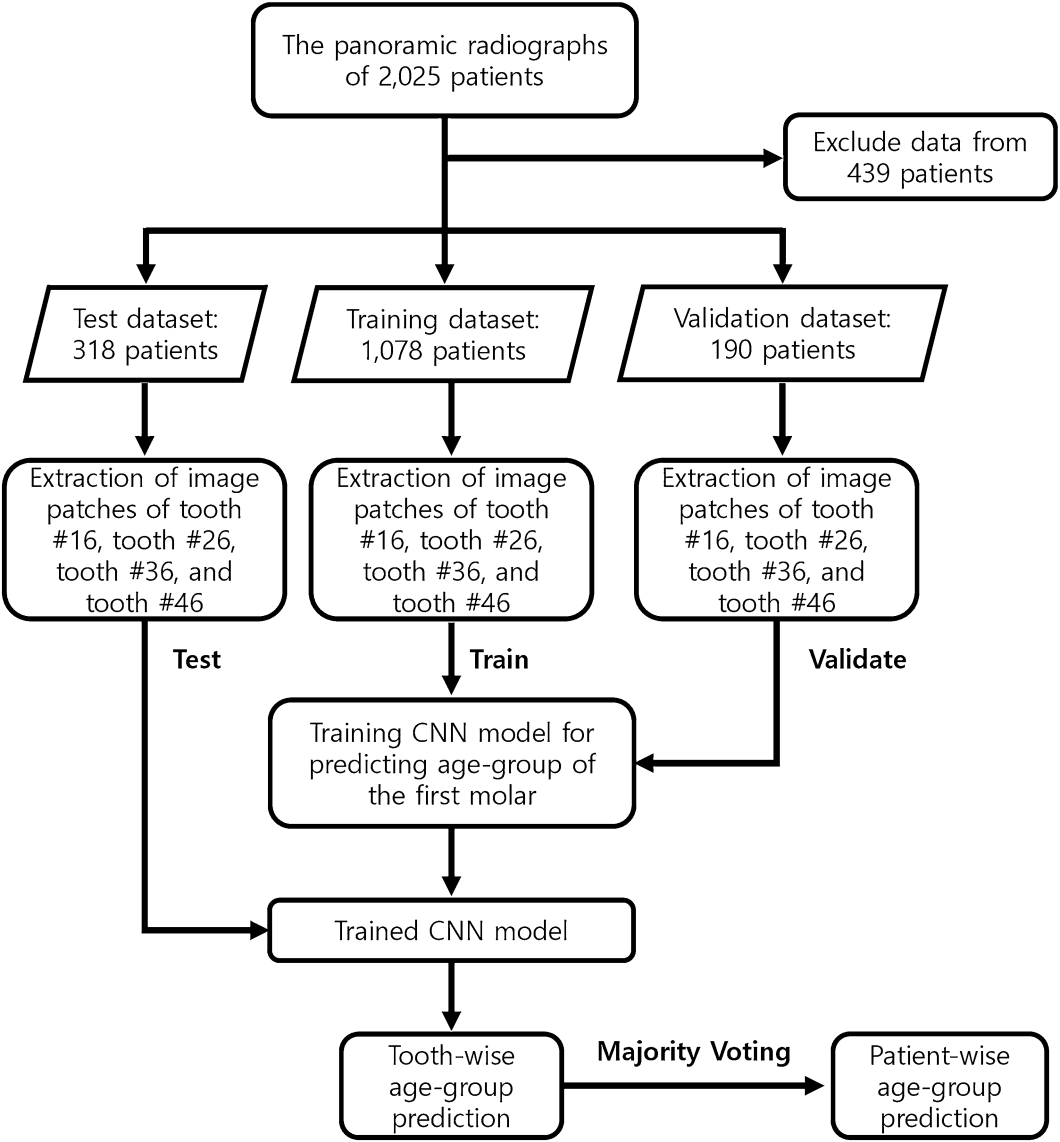
Overall workflow.

The overall workflow of this study is demonstrated in Fig. 1. Panoramic radiographs were collected from 2025 patients who consecutively visited Kyung Hee University Dental Hospital between December 1, 2018, and January 31, 2019. In the panoramic radiograph dataset, only images of people with four intact first molars (#16, #26, #36, and #46) were included, and if any of the four first molars had been lost or replaced with a dental prosthesis, the images were excluded. Non-standardized or low-resolution radiographs were also excluded from the study. Thus, 439 images were excluded from the study, and a total of 1586 patients were included. The 1586 patients were distributed in different age groups according to their legal age as follows: 199 patients aged 0 to 10 y, 197 patients aged 10 to 19 y, 545 patients aged 20 to 29 y, 267 patients aged 30 to 39 y, 182 patients aged 40 to 49 y, 65 patients aged 50 to 59 y, and 131 patients aged greater than 60 y. The number of patients in the young adult group (aged 20 to 29 y) was the highest, and the number of patients aged greater than 60 y was the lowest. The images of 1,586 patients were divided into training, validation, and test datasets according to the following ratio: training:validation:test = 1078:190:318. This division ratio was applied equally to patients across all age groups. With this division ratio, we performed five-fold cross-validation to develop and evaluate the model.

This study was conducted as a preliminary study on an AI-based approach for the estimation of an age group, rather than an estimation of the exact age. For clarity of classification through panoramic radiography, the participants were divided into three age groups: children and adolescents (ages 0–19), young adults (ages 20–49), and older adults (age > 50 years). First, for a wide range of age determination (20 y intervals, three groups in total), the accuracy of the AI model was analyzed. The young adults’ group, which had the largest number of samples, was further divided into three subgroups (ages 20–29, ages 30–39, and ages 40–49). Therefore, we compared the results obtained by dividing the participants into three age groups, and the results obtained by subdividing the young adults into further groups, thereby generating a total of five age groups. In other words, possible changes in estimation accuracy with an increasing number of predicted groups and differences in subdivided groups within young adults were also investigated. The accuracy for estimating the age group for each tooth and for each individual was determined.

Informed consent was obtained from all participants. This study was approved by the Institutional Review Board (IRB) of the Kyung Hee University Dental Hospital and was carried out in accordance with relevant guidelines and regulations.

### Tooth-wise age-group prediction with CNN model

As the first molar is considered to be the most reliable tooth for estimating dental age^15,22^, we selected it for developing a CNN model for the age-group determination. From a panoramic radiograph of each patient, image patches of teeth #16, #26, #36, and #46 were manually extracted. The goal of extracting image patches from the panoramic radiographs was to include complete contours of the teeth. As a result, a total of 4,312 image patches of first molars were collected from 1078 patients for training, and every tooth patch was resized into a fixed size of 151 × 112. To minimize any unnecessary variance in the dataset and to improve the performance of the model, the dataset was augmented^23^, and we used the following selective data augmentation techniques: the tooth images were flipped left and right, upside down, rotated, and reversed by 90°.

The residual deep neural network with 152 layers (ResNet-152)^23^ was trained to predict the age-group tooth-wise, that is, for each tooth. The weights of the network were initialized using pre-trained weights from ImageNet dataset^24^. Then, the entire network was fine-tuned for the target age-group estimation problem. Although the ImageNet base dataset does not include teeth images, various studies have shown that the fine-tuning of a pre-trained network with several images of ImageNet helps improve the performance in disease-related problem learning using medical images^25–27^. The network was trained using the cross-entropy loss function and adaptive moment estimation (Adam) optimizer^28^ with a learning rate of 1e-5 and a batch size of 32. We trained the network for 40,000 iterations and validated it using validation data every 1000 iterations with a classification accuracy metric to determine whether to stop the training. The network showing the highest validation accuracy was selected as the final model and used for testing.

### Patient-wise age-group prediction using majority voting

In the field of forensic dental medicine, age estimation is meaningful at the individual level rather than at the tooth level. Therefore, tooth-wise prediction results obtained from the CNN model based on the four first molar images were ensembled using a majority voting method.

**Figure 2:**
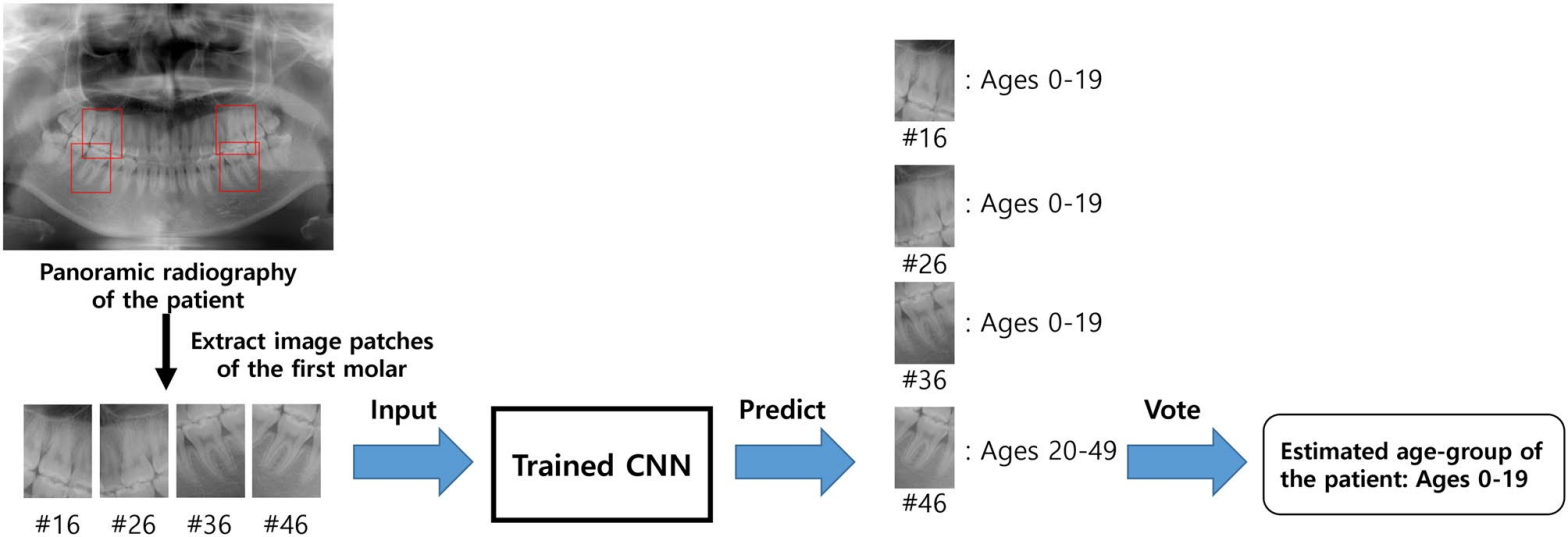
Classification of a panoramic dental X-ray using learned networks to predict age-group of the patient.

The overall test process for predicting a patient-wise age group is illustrated in Fig. 2. First, four image patches of the first molars were extracted from the panoramic radiograph of a patient. Then, each tooth image patch was feed-forwarded to the trained CNN model, and the resulting softmax scores for every age-group class were obtained. The softmax score of each age group represents the probability value that CNNs predict the tooth to the corresponding age group, and the accuracy of the classification task^29^. The age group with the highest softmax score was selected as the prediction result for the age group of each tooth. Finally, among the age-group prediction results of four individual teeth of one person, the age group with the highest number of votes was selected as the patient age group.

### Performance metrics and methodology

We evaluated the age-group estimation performance using the area under curve (AUC) and classification accuracy. For AUC, we constructed a receiver operating characteristic (ROC) curve for each age group using the one versus all strategy, in which classification is performed using binary labels: for each age group, a positive class for the target group, and a negative class for the remaining groups. From the ROC curve of each age group, the corresponding AUC score was calculated. For classification accuracy, we calculated the ratio of the number of correctly estimated patients to the total number of tested patients. Along with the classification accuracy, we also report the corresponding confusion matrix.

**Figure 3:**
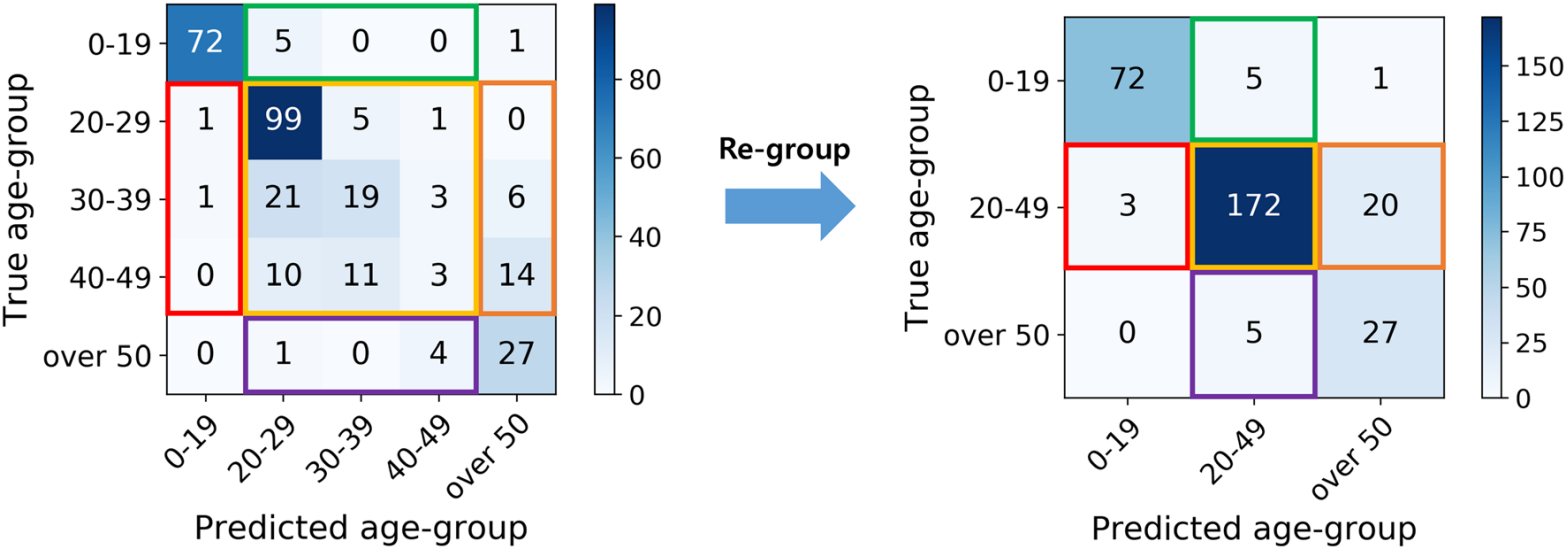
Conversion of the age-group prediction result of the five age-groups to the three age-groups.

As mentioned earlier, CNN models were trained to estimate age groups for two different sets: three age groups and five age groups. Different CNN models were trained for three age groups and five age groups with three and five nodes in their output layers, respectively. Because the numbers of age groups predicted by these CNNs differ, it is impossible to compare the performance metrics of these two CNNs directly. To compare the classification accuracies of the two CNNs directly, patients predicted as young adults in the five-age-group set (ages 20 to 29, 30 to 39, and 40 to 49 y) were merged into the young adult class in the three-age-groups set (ages 20 to 49 y), as shown in Fig. 3. In other words, the prediction results for the five age groups were regrouped into three age groups, allowing the classification accuracies of the two CNNs to be compared. Similarly, for regrouping the AUC results of the five age groups, softmax scores of the CNN model for the ages 20–29, 30–39, and 40–49 of the five age groups were merged by summation to determine the softmax score of ages 20–49 of the three age groups.

### Visual analysis of the learned representation in CNNs

Analyzing the learned features from CNNs can provide a deeper understanding of the most informative region by CNN models in distinguishing age groups. To verify the usefulness and feasibility of this AI-based age-group estimation method, we visualized the learned representation of the trained CNN model using a heatmap image using a gradient-weighted class activation mapping (Grad-CAM) algorithm^29^. For each class *c* in the CNN, the Grad-CAM ${L}_{\mathrm{Grad}-\mathrm{CAM}}^{c}$ is calculated with a linear combination of the *k*-th feature maps of the convolutional layer A^*k*^ and the importance weight ${\alpha }_{K}^{c}$, followed by ReLU activation:

$${L}_{\mathrm{Grad}-\mathrm{CAM}}^{c } =\mathrm{ReLU }(\sum_{K}{\alpha }_{k}^{c }{A}^{k})$$

The weight parameter ${\alpha }_{K}^{c}$ is calculated by the average of the gradient of the score of class c with respect to *A*^*k*^, so that it represents the effect of feature map *k* on target class *c*. Therefore, each pixel value of Grad-CAM ${L}_{\mathrm{Grad}-\mathrm{CAM}}^{c}$ represents the feature sensitivity to changes in the score of class c, with a large value indicating the importance of the pixel for predicting target class *c*. We explored the regions with large Grad-CAM values on a tooth to investigate which parts of the teeth trained CNNs were recognized as more important than others when classifying the age group of a tooth.

### Statistical analysis and evaluation

ROC curves and the AUC were used to investigate system performance. In addition, we compared the age-group prediction results of CNNs trained for predicting two different groupings: three age groups and five age groups. All five age-group prediction results were re-grouped into the three age-group prediction results, and their mean prediction accuracies were compared. To compare the mean values, the unequal variance t-test was performed using the Python package Scipy version 1.4.0. A two-tailed *p* value < 0.05 was considered statistically significant. For a deeper understanding of the learned regions of the model, we generated a heat map with class activation mapping^30^. This map visually highlights the tooth regions of the x-ray patches from the panoramic radiographs that are most informative for distinguishing the age group.

## Results

### Tooth-wise age-group prediction using CNNs

The accuracy of tooth-wise age-group prediction using a CNN model is shown in the first column of Table 1. The overall accuracy when using individual teeth ranges between 87.04 and 88.33%. However, the accuracy of tooth-wise age-group prediction did not differ significantly according to the location of the first molar and the number of age-groups. The high accuracy of each tooth showed that the image of any individual first molar is sufficient for determining the age group of a person. For the networks trained for the three age groups, teeth #36 and #46 were slightly more useful, with accuracies of 88.33 ± 0.94% and 88.18 ± 0.71%, respectively, than the networks for #16 and #26 (87.36 ± 0.53% and 87.39 ± 0.48%, respectively). On the contrary, for networks trained to predict five groups, #16 and #26 were determined to be more useful for our CNNs with accuracies of 87.76 ± 0.67% and 88.16 ± 0.71%, respectively, compared with accuracies of 87.04 ± 0.81% and 87.04 ± 0.71% for teeth #36 and #46, respectively. The results partly confirm the hypothesis of this study that the information in different first molar teeth and in different regimes of each tooth can be used in diverse ways, which was the motivation for applying AI algorithms. Interestingly, in tooth-wise prediction, no particular tooth was always the most useful for all target settings used.

**Table 1:** Age-group estimation accuracy of tooth-wise and patient wise predictions.

	Tooth-wise accuracy		Patient-wise accuracy		Combining the prediction results	
	Tooth	Accuracy (%)	Accuracy (%)	*p* value	Accuracy (%)	*p* value
Three age-group prediction	#16	87.36 $\pm$ 0.53	**89.05** $\pm$ **0.68**	0.117	**90.37** $\pm$ **0.93**	0.043*
	#26	87.39 $\pm$ 0.48		0.114		0.043*
	#36	88.33 $\pm$ 0.94		0.595		0.205
	#46	88.18 $\pm$ 0.71		0.452		0.136
Five age-group prediction	#16	87.76 $\pm$ 0.67	**89.21** $\pm$ **0.44**	0.150		0.079
	#26	88.16 $\pm$ 0.71		0.298		0.132
	#36	87.04 $\pm$ 0.81		0.079		0.043*
	#46	87.36 $\pm$ 0.53		0.055		0.036*

The results were obtained using unequal variance t-tests. A two-tailed *p* value < 0.05 was considered statistically significant. *p value < 0.05.

Significant variables and results shown in bold text.

### Visualization of Grad-CAM for tooth-wise age-group prediction

**Figure 4:**
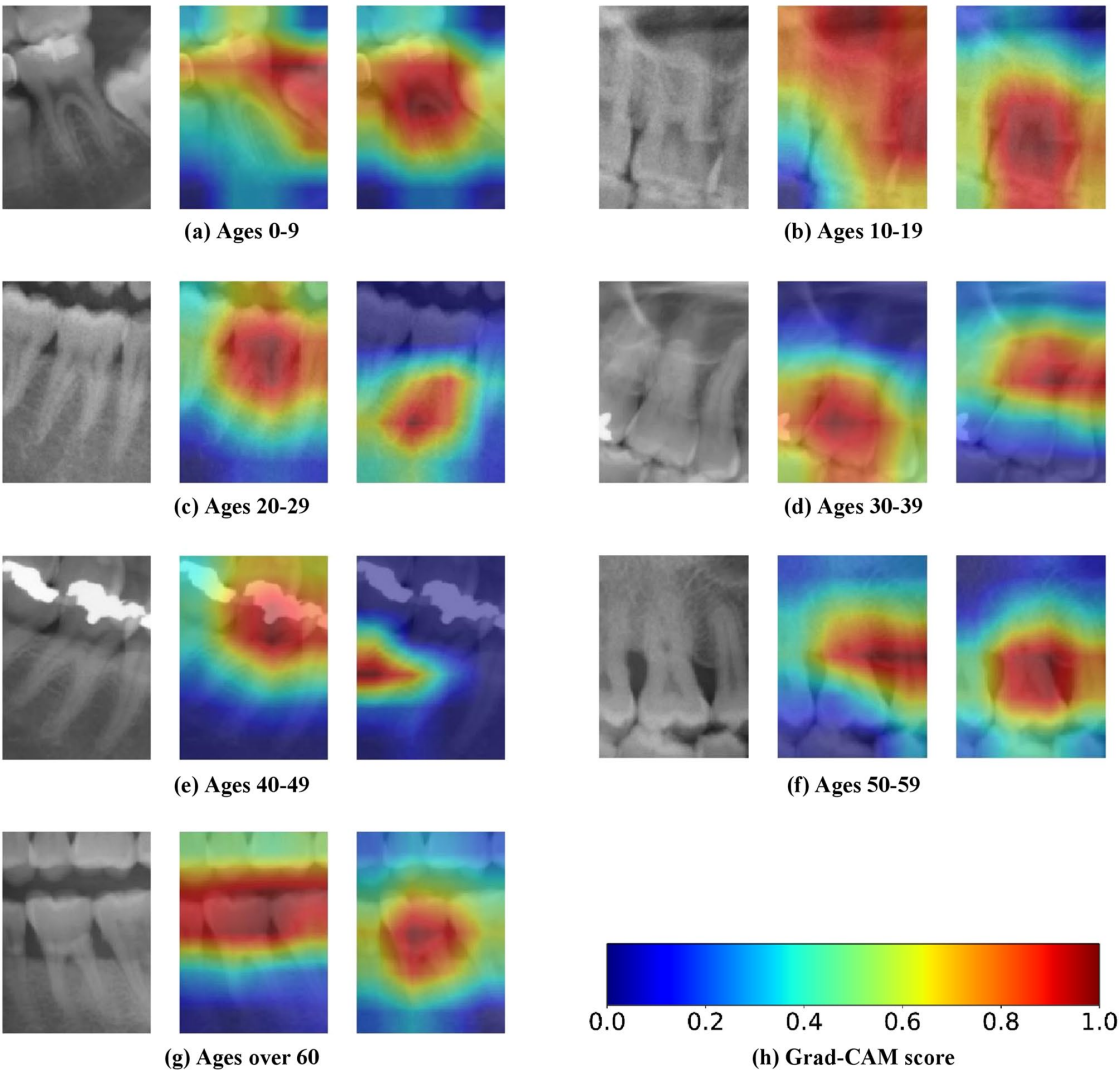
Example of correctly classified teeth patches and their Grad-CAM results. The figures show the original molar image (left), Grad-CAM for three age-group prediction (middle) and Grad-CAM for five age-group prediction (right). Note that the softmax score for every first molar image in the figure is above 0.99.

To investigate which visual features of the teeth are important for CNNs, we visualized the learned representation in CNN. Figure 4 shows the heatmaps of the Grad-CAMs for the given input teeth images and represents the feature sensitivity of the CNNs for tooth-wise prediction. Every first molar X-ray image extracted from the panoramic radiograph in Fig. 4 was used to predict the correct age group with CNN with a softmax confidence of more than 0.99. For each original X-ray of an individual tooth, the heatmap for the three age-group network (middle) and the heatmap for the five age-group network (right) are shown. In each heatmap image, the important regime for the CNN decision is marked in red, and the less important area is marked in blue.

Interestingly, even if the same X-ray image is given as input, it can be seen that the important parts for CNNs are different depending on the age grouping. For ages 0–9, when classifying three age groups, the relationship with the second molar and the degree of eruption of the second molar were recognized. In contrast, when classifying five age groups, the pulp of the first molar was recognized as the most important feature (Fig. 4a). Ages 10–19 were recognized by alveolar bone condition and maxillary sinus when classifying the three age groups. In contrast, in the classification using five age groups, the pulp of the first molar was recognized (Fig. 4b). For ages 20–29, the pulp of the first molar was recognized when classifying the three age groups. The periapical area of the first molar was recognized when classifying the five age groups (Fig. 4c). For ages 30–39, the pulp of the first molar was recognized when classifying the three age groups.

The interdental space between the first molar and the second molar was recognized in the five age-group classifications (Fig. 4d). For ages 40–49, the pulp of the first molar was recognized when classifying the three age groups. The inter-dental space and alveolar bone level between the first molar and the second molar were recognized for five age groups (Fig. 4e). For ages 50–59 years, the inter-dental space and the alveolar bone level between the first molar and the second molar were recognized when classifying the three age groups. Alveolar bone levels were observed in the five age groups (Fig. 4f). For patients aged > 60 years, occlusal levels of the teeth were recognized when classifying the three age groups. The pulp of the first molar was recognized in the five age-group classifications (Fig. 4g). The learned features and focused regions of the panoramic radiographic images by CNNs varied according to the location of the first molar, the age, and the criteria for age grouping. Overall, diverse visual features were recognized for determining correct targets, depending on age, and tooth location of the input teeth images.

### Patient-wise age-group prediction using majority voting

The accuracy of patient-wise age-group prediction with this CNN model is shown in Table 1 (the second column). The accuracies of patient-wise prediction for the three age-grouping and the five age-grouping were 89.05 ± 0.68% and 89.21 ± 0.54% (*p* value = 0.83), respectively. Similar to the tooth-wise age-group prediction, there was no significant difference in classification accuracy between the two age groups. However, the average accuracy of the patient-wise age-group prediction obtained using majority voting is higher than the maximum accuracy for an individual tooth for both the three—and five-age-group predictions.

**Figure 5:**
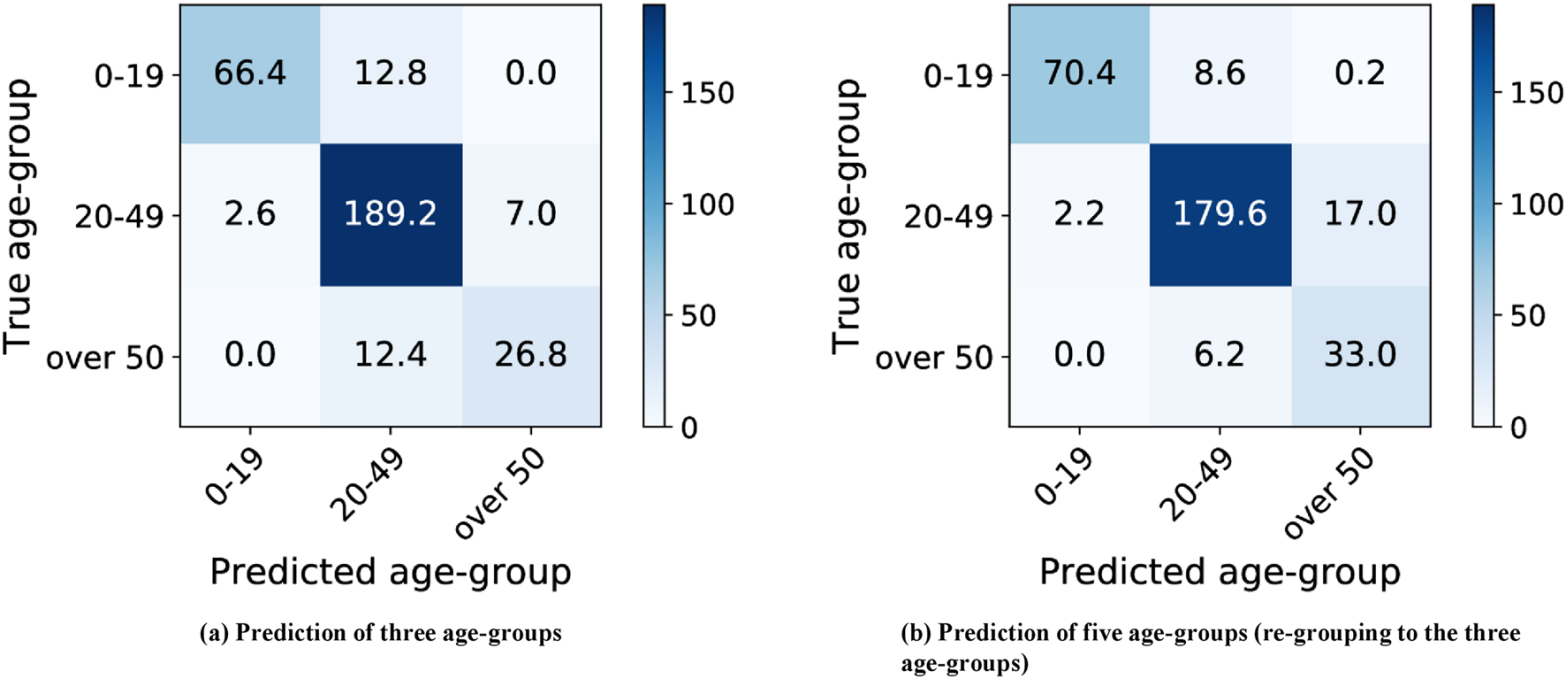
Confusion matrix of the age-group estimation results of the predictions for the three age-groups and five age-groups. In (**a**), the network trained for predicting three age-groups. In (**b**), the network trained for predicting five age-groups is used for three age-group prediction. Each element of the confusion matrix represents the average number of patients over five-fold cross validation.

Figure 5 shows the corresponding confusion matrix of the three age-group predictions (Fig. 5a) and five age-group predictions (Fig. 5b). Each element of the confusion matrix represents the average number of patients over five-fold cross-validation. Although the accuracies do not significantly differ, the corresponding confusion matrices show different trends in predictions for different age groups. When we compare the second columns of Fig. 5a,b, the network trained to predict using the three age groups shows a higher tendency to classify teeth as being of ages 20–49 than the network trained for five age-group predictions. From Fig. 5, the trained network for the three-age-group prediction can better discriminate adults and elderly, and the trained network for five age-group prediction discriminates children, adolescents, and adults better.

**Figure 6:**
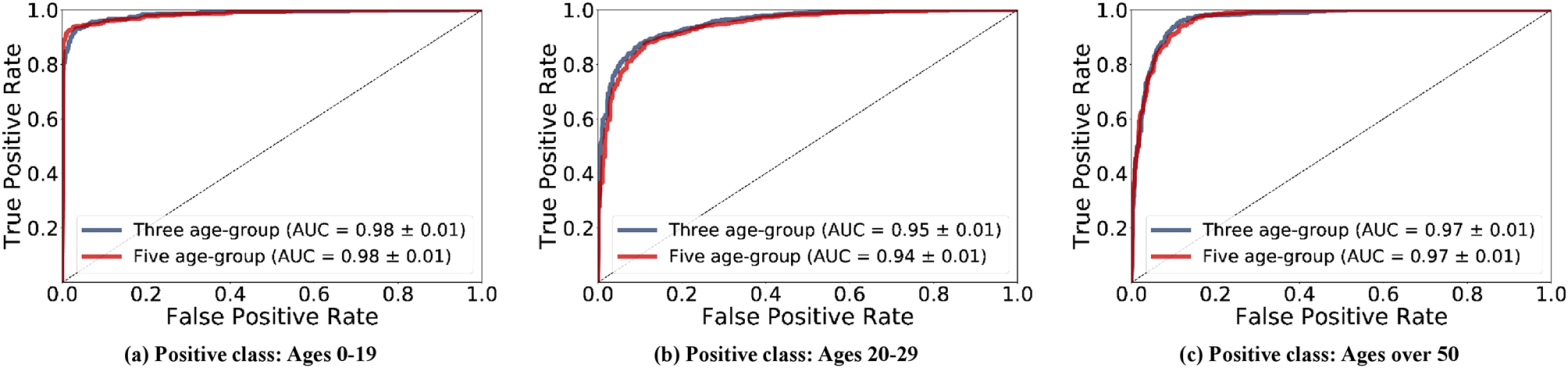
A comparison of average ROC curves over five-fold cross validation between the networks to obtain predictions for three age-groups and five age-groups. Each curve is obtained by binary classification to discriminate one age-group from others. The network that learned to predict five age-groups is used to predict the three age-groups. The AUC of each age-group shows (a) ages 0–19: Three-age group = 0.98 ± 0.01 and five-age group = 0.98 ± 0.01 (*p* value = 0.97), (b) ages 20–49: three-age group = 0.95 ± 0.01 and five-age group = 0.94 ± 0.01 (*p* value = 0.12), (**c**) ages over 50: three-age group = 0.97 ± 0.01 and five-age group = 0.97 ± 0.01 (*p* value = 0.98).

Figure 6 shows the patient-wise age-group prediction performance of each age group using the ROC curves and AUC scores for the discrimination of each group. AUC achieved high scores for all age groups, ranging from 0.94 to 0.98. The high AUC scores indicate that the images of first molars #16, #26, #36, and #46 contain age-related visual features, and that the CNNs successfully exploited those features to predict the correct age groups. In addition, there were non-significant *p-*values between the AUC scores of the three age-group predictions and five age-group predictions (*p* values of ages 0–19: 0.97, ages 20–49: 0.12, and ages over 50: 0.98), which indicates that different age groups do not significantly affect the classification ability and accuracy scores.

### Combining the prediction results of different age groupings

We further extended the majority voting method to patient-wise predictions by combining the tooth-wise predictions of the three age groups and the five age groups together. As a result, a total of eight tooth-wise age-group predictions, based on four predictions from three age groups and four predictions from five age groups, were used for majority voting. Patient-wise age-group prediction is determined as the most frequently predicted age group among the eight tooth-wise predictions. As a result of extended majority voting, the average accuracy of patient-wise prediction is further improved to 90.37% (the third column of Table 1). In addition, lower p-values between the extended majority voting and each individual tooth-wise prediction show that majority voting using eight tooth-wise predictions significantly improves accuracy compared with majority voting using only four tooth-wise predictions.

## Discussion

Age determination is important in the forensic field, not only for the identification of the deceased, but also for living individuals. Among the various biological markers for age determination, teeth are a crucial indicator of aging^31^. Methods for age determination are mostly based on X-rays, and assess crown formation, mineralization of a tooth, root growth and apex maturation, and order of eruption of the teeth into the mouth^32,33^. While these various dental age estimation methods have been confirmed for children and adolescents, there are few reliable methods with low error rates and high reliability for adults and the elderly. In addition, these conventional methods focus on one or two characteristics in panoramic radiographic images, and do not consider other parts of the images except for the parts with attention^34,35^. Image classification using a CNN model is the task of categorizing images into one of several predefined classes using computer vision^36^. Overall, we present a breakthrough dental age estimation method with high accuracy, as patient-wise estimation accuracy was 89.05 to 90.27%, which automatically looks at multiple features in one of the first molar X-ray images. The AI-based CNN model with majority voting proposed here can be used in age group estimation in forensic dentistry across all age groups.

Furthermore, important features for tooth-wise prediction were highlighted through Grad-CAM visualization. Grad-CAM can produce a coarse localization map highlighting the important regions in an image that aids prediction and wherein the algorithm is focused on^37^. In our CNN model, CNNs learn from various other regions, including a lowered occlusal surface due to tooth attrition, decreased alveolar bone level, and increased interdental space, however, usually from the pulp of the tooth, depending on the shape and location of the first molar when classifying the age group. Tooth pulp was considered an important visual feature in determining age^14,38^. In addition, loss of alveolar bone is associated with aging^10^. However, unlike human observations and conventional dental age estimation methods that use only specific individual features, the AI-based age-group estimation model has the overwhelming strength of using comprehensive and automatic judgment of various anatomical factors^39^. In this respect, Grad-CAM is very useful, but as this is the first study in which it is applied to tooth X-ray images obtained from panoramic radiographs, further research on dental X-ray applications is needed.

Although the same X-ray image patches extracted from panoramic radiographs are given as inputs, the recognized regions from the CNNs differ depending on the age grouping while training the network. This can be observed for every age group that CNNs were trained with, the three age group and five age-group predictions focused on completely different regions of the same X-ray image patches. In training CNNs, even if the same data are given, different parts of the data can be learned depending on the task^40^. With CNNs, multi-task learning has become possible, and this method improves the accuracy of a target task by simultaneously learning targets and by recognizing related adjacent structures^41^. Thus, this characteristic of an AI-based age-group estimation model is a very useful aspect in forensic dental medicine that can generally be well applied across various people and age groups.

What is the meaning of improved patient-wise prediction accuracy via majority voting? We obtained patient-wise age-group predictions using the majority voting method of the individual tooth-wise age-group predictions. According to Karaarslan et al.^42^, age estimation by evaluating panoramic radiographs with the naked eye was the most accurate for the first decade of life (89.6%) and the lowest for the fourth decade of life (41.7%). However, with the AI-based approach, we achieved high numerical accuracy for all age groups. The results of patient-wise predictions show that the accuracy of patient-wise prediction increases as the number of tooth-wise predictions used for majority voting increases. Age estimation by humans may be focused on the same features for a single individual, even if several teeth or anatomical features are used^15,43^. Of course, the improved accuracy using the four first molars indicates that CNNs learn partially independent information from the four different first molars, even for only one person. The improved performance upon majority voting may indicate that each individual prediction provides partially independent information^44,45^. Even within an individual, teeth in different locations and of different sizes and shapes can provide independent information.

Furthermore, as a result of extending the ensemble of eight presentations, the average accuracy was improved to 90.37% compared with the accuracy obtained by an ensemble of only four predictions with 89.05% for the three age groups and 89.21% for the five age groups. An ensemble method is a meta-algorithm that combines several deep learning techniques into one predictive model, and aims to decrease variance, bias, and improve predictions^46^. Moreover, as the amount of data that can be learned increases and varies, the accuracy of an AI-based model is bound to increase by^47^. Further improved accuracy was obtained when combining the predictions of different age groupings, which indicates that CNNs learn different information from the same teeth when the age grouping is varied during training. Our results from majority voting were consistent with the visual analysis. As a result, we confirm the following two properties of CNNs for predicting the age groups of teeth: CNNs learn different features from each individual tooth, and CNNs learn different features from the same tooth depending on the age grouping used for learning.

It is worth reporting that this is the first attempt to differentiate age groups using CNN models for teeth. Unfortunately, we only targeted the first four molars of an individual. In addition, the number of subjects was limited because patients had to actually have undergone panoramic radiographs. Another limitation is that the data are biased toward young adults (20 to 49 y) because patients in that age group visited the hospital frequently during the data collection period. As follow-up research, we will include additional teeth and adjacent structures of a large number of subjects for deep learning, which can further increase the age estimation performance. Through neural network ensembles, the estimation accuracy was increased when eight predictions were synthesized, and age-group estimation was performed. Therefore, as the prediction number increases, the accuracy may increase, so it is necessary to further investigate the targeting of multiple teeth or the simultaneous analysis of various anatomical factors of the entire orofacial area.

## Conclusion

In this study, we showed that CNNs are well suited to the task of estimating the age groups of the maxillary and mandibular first molars. The high AUC score and classification accuracy for age-group estimation implies that the first molars contain age-related visual features. A visual investigation using Grad-CAM reveals that CNNs learn a set of age-related holistic features and find important features in the shape of the target tooth. In addition, the Grad-CAM visualization also shows that CNNs trained with different age-group resolutions learn to not necessarily use the same visual features on identical teeth. The boosted performance in ensemble experiments supports the fact that CNNs learn diverse information from different first molars and varying the age-group resolution leads CNNs to learn partially independent information across each age-group resolution. Although this study used only the four first molars, the ensemble result suggests that the age-group estimation performance can be further improved with the inclusion of additional teeth of a large number of subjects with age-related information to the learning of CNNs in the future.

## Data Availability

The datasets generated and/or analyzed during this study are available from the corresponding author upon reasonable request. Because patient consent is required for data disclosure, we may disclose data conditionally through internal discussion and the Institutional Review Board (IRB) of the Kyung Hee University Dental Hospital.

## Code availability

The code underlying this study is available at: https://github.com/nonstop1962/pytorch-dental_age_estimation.

## References

[1] Sajid M, Taj IA, Bajwa UI, Ratyal NI (2018). Facial asymmetry-based age group estimation: role in recognizing age-separated face images. J. Forensic Sci..

[2] Sykes L, Bhayat A, Bernitz H (2017). The effects of the refugee crisis on age estimation analysis over the past 10 years: a 16-country survey. Int. J. Environ. Res. Public Health.

[3] Ren X (2019). Regression convolutional neural network for automated pediatric bone age assessment from hand radiograph. IEEE J. Biomed. Health Inform..

[4] Mihail RP, Liang G, Jacobs N (2019). Automatic hand skeletal shape estimation from radiographs. IEEE Trans. Nanobiosci..

[5] Manzoor Mughal A, Hassan N, Ahmed A (2014). Bone age assessment methods: a critical review. Pak. J. Med. Sci..

[6] Rozylo-Kalinowska I, Kolasa-Raczka A, Kalinowski P (2011). Relationship between dental age according to Demirjian and cervical vertebrae maturity in Polish children. Eur. J. Orthod..

[7] Moorrees CF, Fanning EA, Hunt EE (1963). Age variation of formation stages for ten permanent teeth. J. Dent. Res..

[8] Cameriere R (2016). Age estimation in children by measurement of open apices in teeth with Bayesian calibration approach. Forensic Sci. Int..

[9] Gotmare SS (2019). The coronal pulp cavity index: a forensic tool for age determination in adults. Dent. Res. J. (Isfahan).

[10] Sarajlić N, Topić B, Brkić H, Alajbeg IZ (2009). Aging quantification on alveolar bone loss. Coll. Antropol..

[11] Ruquet M, Saliba-Serre B, Tardivo D, Foti B (2015). Estimation of age using alveolar bone loss: forensic and anthropological applications. J. Forensic Sci..

[12] Koh KK (2017). Age estimation from structural changes of teeth and buccal alveolar bone level. J. Forensic Leg. Med..

[13] Cameriere R, Ferrante L, Cingolani M (2004). Precision and reliability of pulp/tooth area ratio (RA) of second molar as indicator of adult age. J. Forensic Sci..

[14] Juneja M, Devi YB, Rakesh N, Juneja S (2014). Age estimation using pulp/tooth area ratio in maxillary canines-A digital image analysis. J. Forensic Dent. Sci..

[15] Shah PH, Venkatesh R (2016). Pulp/tooth ratio of mandibular first and second molars on panoramic radiographs: an aid for forensic age estimation. J. Forensic Dent. Sci..

[16] Huttner EA, Machado DC, de Oliveira RB, Antunes AG, Hebling E (2009). Effects of human aging on periodontal tissues. Special Care Dent..

[17] Chen H (2019). A deep learning approach to automatic teeth detection and numbering based on object detection in dental periapical films. Sci. Rep..

[18] Vinayahalingam S, Xi T, Bergé S, Maal T, de Jong G (2019). Automated detection of third molars and mandibular nerve by deep learning. Sci. Rep..

[19] Krois J (2019). Deep learning for the radiographic detection of periodontal bone loss. Sci. Rep..

[20] Demirjian A, Goldstein H, Tanner JM (1973). A new system of dental age assessment. Hum. Biol..

[21] Merdietio-Boedi R (2020). Effect of lower third molar segmentations on automated tooth development staging using a convolutional neural network. J. Forensic Sci..

[22] Mathew DG (2013). Adult forensic age estimation using mandibular first molar radiographs: a novel technique. J. Forensic Dent. Sci..

[23] He, K., Zhang, X., Ren, S. & Sun, J. Deep residual learning for image recognition. In *2016 IEEE Conference on Computer Vision and Pattern Recognition (CVPR), Las Vegas, NV*, 2016, 770–778, 10.1109/CVPR.2016.90.

[24] Russakovsky O, Deng J, Su H (2015). ImageNet large scale visual recognition challenge. Int. J. Comput. Vis..

[25] Tajbakhsh N (2016). Convolutional neural networks for medical image analysis: full training or fine tuning?. IEEE Trans. Med. Imaging.

[26] Shin H-C (2016). Deep convolutional neural networks for computer-aided detection: CNN architectures, dataset characteristics and transfer learning. IEEE Trans. Med. Imaging.

[27] Chang, J., Yu, J., Han, T., Chang, H-J., & Park, E. (2017). A method for classifying medical images using transfer learning: a pilot study on histopathology of breast cancer. In *2017 IEEE 19th International Conference on e-Health Networking, Applications and Services, Healthcom 2017* (pp. 1–4). (2017 IEEE 19th International Conference on e-Health Networking, Applications and Services, Healthcom 2017; Vol. 2017-December). Institute of Electrical and Electronics Engineers Inc. 10.1109/HealthCom.2017.8210843

[28] Dubey SR (2019). diffGrad: an optimization method for convolutional neural networks. IEEE Trans. Neural Netw. Learn. Syst..

[29] Iizuka T, Fukasawa M, Kameyama M (2019). Deep-learning-based imaging-classification identified cingulate island sign in dementia with Lewy bodies. Sci. Rep..

[30] Ohannessian R (2017). Heat map for data visualization in infection control epidemiology: an application describing the relationship between hospital-acquired infections, Simplified Acute Physiological Score II, and length of stay in adult intensive care units. Am. J. Infect. Control.

[31] Jia L, Zhang W, Chen X (2017). Common methods of biological age estimation. Clin. Interv. Aging.

[32] Mohammed RB (2014). Digital radiographic evaluation of mandibular third molar for age estimation in young adults and adolescents of South Indian population using modified Demirjian's method. J. Forensic Dent. Sci..

[33] Ilayaraja V (2018). Digitized morphometric analysis using maxillary canine and mandibular first molar for age estimation in South Indian population. Open Dent. J..

[34] Garamendi PM, Landa MI, Botella MC, Alemán I (2011). Forensic age estimation on digital X-ray images: medial epiphyses of the clavicle and first rib ossification in relation to chronological age. J. Forensic Sci..

[35] Limdiwala PG, Shah JS (2013). Age estimation by using dental radiographs. J. Forensic Dent. Sci..

[36] Rawat W, Wang Z (2017). Deep convolutional neural networks for image classification: a comprehensive review. Neural Comput..

[37] Tang Z (2019). Interpretable classification of Alzheimer's disease pathologies with a convolutional neural network pipeline. Nat. Commun..

[38] Krishan K, Kanchan T, Garg AK (2015). Dental evidence in forensic identification—an overview, methodology and present status. Open Dent. J..

[39] Liao H, Yan Y, Dai W, Fan P (2018). Age estimation of face images based on CNN and divide-and-rule strategy. Math. Probl. Eng..

[40] Baltruschat IM, Nickisch H, Grass M, Knopp T, Saalbach A (2019). Comparison of deep learning approaches for multi-label chest x-ray classification. Sci. Rep..

[41] Sakata A, Takemura N, Yagi Y (2019). Gait-based age estimation using multi-stage convolutional neural network. IPSJ Trans. Comput. Vis. Appl..

[42] Karaarslan B, Karaarslan ES, Ozsevik AS, Ertas E (2010). Age estimation for dental patients using orthopantomographs. Eur. J. Dent..

[43] Jain S (2017). Tooth coronal index and pulp/tooth ratio in dental age estimation on digital panoramic radiographs-A comparative study. Forensic Sci. Int..

[44] Hansen LK, Salamon P (1990). Neural network ensembles. IEEE Trans. Pattern Anal. Mach. Intell..

[45] Irvine N, Nugent C, Zhang S, Wang H, Ng WWY (2019). Neural network ensembles for sensor-based human activity recognition within smart environments. Sensors (Basel).

[46] Abuassba AOM, Zhang D, Luo X, Shaheryar A, Ali H (2017). Improving classification performance through an advanced ensemble based heterogeneous extreme learning machines. Comput. Intell. Neurosci..

[47] Schmidt J, Marques MRG, Botti S, Marques MAL (2019). Recent advances and applications of machine learning in solid-state materials science. Comput. Mater..

