# Author Correction: Age-group determination of living individuals using first molar images based on artificial intelligence

**DOI:** 10.1038/s41598-022-06543-7

**Published:** 2022-02-07

**Authors:** Seunghyeon Kim, Yeon‑Hee Lee, Yung‑Kyun Noh, Frank C. Park, Q.‑Schick Auh

**Affiliations:** 1 Robotics Laboratory, Department of Mechanical and Aerospace Engineering, Seoul National University, Seoul, Korea; 2 Department of Orofacial Pain and Oral Medicine, Kyung Hee University Dental Hospital, #26 Kyunghee‑daero, Dongdaemun‑gu, Seoul, 02447 Korea; 3 Department of Computer Science, Hanyang University, Seoul, Korea

Correction to: *Scientific Reports* 10.1038/s41598-020-80182-8, published online 13 January 2021

The original version of this Article did not include the link to the code repository. This is now included in the Code Availability section which reads:

The code underlying this study is available at: https://github.com/nonstop1962/pytorch-dental_age_estimation.

The Article has been corrected.

